# Polytrauma im DACH-Raum: Vergleich von Traumaregisterdaten aus Deutschland, Österreich und der Schweiz

**DOI:** 10.1007/s00113-025-01674-8

**Published:** 2026-01-09

**Authors:** Houmam Anees, Thaqif El Khassawna, Christian Heiß, Christoph Biehl

**Affiliations:** 1 Klinik und Poliklinik für Unfall‑, Hand-, und Wiederherstellungschirurgie, Universitätsklinikum Gießen und Marburg, Standort Gießen, Justus-Liebig-Universität Gießen, Rudolf-Buchheim-Str. 7, 35385 Gießen, Deutschland; 2 Labor für Experimentelle Unfallchirurgie, Justus-Liebig-Universität Gießen, 35392 Gießen, Deutschland; 3 School of Pharmacy, The University of Jordan, 11942 Amman, Jordanien; 4 Department of Orthopaedics and Traumatology, University of Biruni, Istanbul, Türkei

**Keywords:** Schwerverletzte, Traumaversorgung, Prähospitale Versorgung, Intensivmedizinische Behandlung, Gesundheitsökonomie, Severely injured patients, Trauma care systems, Prehospital emergency care, Intensive care management, Health economic analysis

## Abstract

**Hintergrund:**

Traumaregister ermöglichen eine systematische Bewertung der Versorgungsqualität schwer verletzter Patienten. Ziel dieser Arbeit ist der Vergleich zentraler epidemiologischer, klinischer und prozessbezogener Parameter aus Deutschland, Österreich und der Schweiz anhand ihrer nationalen Traumaregister.

**Methodik:**

Es wurde eine narrative, deskriptive Analyse der aktuell verfügbaren Registerdaten durchgeführt. Für Deutschland (TR-DGU, 2023) und Österreich (TR-ÖGU, 2023) wurden ausschließlich Daten des Basiskollektivs verwendet [1, 2]. Für die Schweiz wurden mangels Jahresberichten die publizierten Daten des Swiss Trauma Registry (STR, 2015–2019; Costa et al.) ausgewertet, die ausschließlich Fälle mit einem ISS ≥ 16 und/oder AIS des Kopfes ≥ 3 umfassen [3, 4]. Aufgrund unterschiedlicher Einschlusskriterien und Zeiträume wurde kein inferenzstatistischer Vergleich durchgeführt.

**Ergebnisse:**

Deutschland dokumentierte 2023 im Basiskollektiv 31.217 Patienten, davon 28.718 primär versorgt (Ø 54,5 Jahre, 69,6 % männlich, Ø ISS 18,5). In der Outcome-Kohorte von 25.208 Patienten lag die Krankenhausmortalität bei 7,4 % [1]. Österreich erfasste im selben Jahr 1060 Fälle (Ø 50,2 Jahre, 73,8 % männlich, Ø ISS 22,6) mit einer Mortalität von 14,7 % [2]. In der Schweiz wurden in den publizierten STR-Analysen zwischen 2015 und 2019 insgesamt 13.222 Patienten erfasst (Ø 58 Jahre, 68 % männlich, Ø ISS 22) mit einer Mortalität von 11,6 % [3, 4]. Während in allen 3 Ländern stumpfe Traumata dominieren (> 90 % in DE/AT, ≈ 94 % geschätzt aus Costa et al.), unterscheiden sich die prähospitalen Strukturen: Deutschland und Österreich setzen auf ein notarztbasiertes System, die Schweiz stärker auf Paramedics. Prozessparameter wie präklinische Intubation, Schockraum-CT oder Transfusionen variieren zwischen den Ländern, zeigen jedoch insgesamt vergleichbare Versorgungsabläufe.

**Schlussfolgerung:**

Die Traumaregister im DACH-Raum zeigen insgesamt vergleichbare Strukturen in der Versorgung schwer verletzter Patienten, unterscheiden sich jedoch in Altersprofil, Fallselektion, Mechanismen und Mortalität. Da für die Schweiz ausschließlich publizierte STR-Analysen verfügbar sind, die nur schwer verletzte Patienten einschließen, ist die Schweizer Kohorte definitionsgemäß schwerer verletzt als die deutschen und österreichischen Basiskollektive. Harmonisierte Registerstrukturen und gemeinsame Auswertungen könnten künftig dazu beitragen, länderübergreifend voneinander zu lernen und die Versorgungsqualität weiter zu verbessern.

**Graphic abstract:**

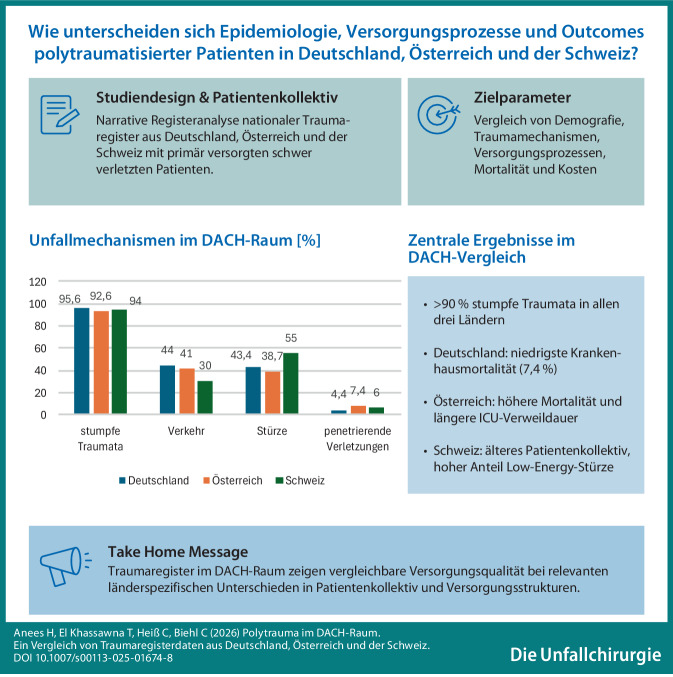

## Abstract (en)

**Background:**

Trauma registries provide essential insights into the quality of care for severely injured patients. This study compares key epidemiological, clinical and process-related parameters from Germany, Austria and Switzerland based on the respective national trauma registry data.

**Methods:**

A narrative descriptive analysis of the most recently available registry data was performed. For Germany (TR-DGU, 2023) and Austria (TR-ÖGU, 2023), only data from the core dataset were included [1, 2]. For Switzerland, in the absence of national annual reports, published analyses of the Swiss Trauma Registry (STR, 2015–2019; Costa et al.) were used, which exclusively include patients with an injury severity score (ISS) ≥ 16 and/or abbreviated injury scale (AIS) score of the head ≥ 3 [3, 4]. Due to differences in inclusion criteria and time periods, no inferential statistical comparisons were conducted.

**Results:**

In 2023 Germany documented 31,217 patients in the core dataset, including 28,718 primary admissions (mean age 54.5 years, 69.6% male, mean ISS 18.5). The in-hospital mortality in the outcome cohort of 25,208 patients was 7.4% [1]. Austria reported 1060 cases in the same year (mean age 50.2 years, 73.8% male, mean ISS 22.6) with a mortality of 14.7% [2]. In Switzerland, the published STR analyses between 2015 and 2019 included a total of 13,222 patients (mean age 58 years, 68% male, mean ISS 22) with a mortality of 11.6% [3, 4]. While blunt trauma dominated in all three countries (> 90% in Germany and Austria, ≈ 94% estimated for Switzerland), prehospital structures differed: Germany and Austria rely on emergency physician-based systems, whereas Switzerland relies more on paramedics. Process parameters such as prehospital intubation, whole-body CT in the trauma room and transfusion rates vary between countries but overall indicate comparable clinical care pathways.

**Conclusion:**

Trauma care in the DACH region is broadly comparable but differs in patient demographics, case selection, trauma mechanisms and mortality. Because the STR data used in this study are derived exclusively from publications including only severely injured patients, the Swiss cohort by definition reflects a more severely injured population than the German and Austrian core datasets. Harmonized registry structures and joint analyses could enhance transnational learning and further improve the quality of care.

## Einleitung

Das Polytrauma stellt nach wie vor eine der größten Herausforderungen in der Akut- und Intensivmedizin dar. Trotz signifikanter Fortschritte in der prähospitalen Versorgung, im Schockraummanagement und in der Intensivtherapie bleibt die Letalität hoch. Ein Polytrauma ist eine der führenden Todesursachen bei jungen Erwachsenen; gleichzeitig nimmt die Zahl geriatrischer Patienten durch Stürze zu [1, 5]. Aktuelle Entwicklungen verdeutlichen, dass Versorgungskonzepte zunehmend an die spezifischen Bedürfnisse älterer Traumapatienten angepasst werden müssen [5].

Die Notwendigkeit, Versorgungsstrukturen international zu vergleichen, wurde bereits Ende der 1990er-Jahre betont. Oestern publizierte 1999 in *Der Unfallchirurg* eine Übersichtsarbeit, in der internationale Unterschiede zwischen Rettungssystemen, Traumazentren und Outcome-Indikatoren hervorgehoben wurden [6]. Ein zentrales Ergebnis dieser Arbeit war die Bedeutung von Traumaregistern wie dem damals noch jungen TraumaRegister DGU® (TR-DGU).

Heute stehen mit dem TR-DGU in Deutschland, dem TR-ÖGU in Österreich und dem STR in der Schweiz 3 leistungsfähige Register zur Verfügung. Bisherige Vergleiche konzentrierten sich jedoch überwiegend auf länderspezifische Ergebnisse; eine integrative Übersicht über den gesamten DACH-Raum fehlt bislang. Ziel der vorliegenden Übersicht ist es, deren aktuelle Daten zusammenzufassen, Gemeinsamkeiten und Unterschiede herauszuarbeiten und ihre Bedeutung für die klinische Versorgung im DACH-Raum zu diskutieren.

## Methodik

Diese Arbeit basiert auf einer narrativen Auswertung der aktuell verfügbaren Traumaregisterdaten aus Deutschland, Österreich und der Schweiz. Für Deutschland und Österreich wurden die Daten des Basiskollektivs der jeweiligen Jahresberichte 2023 verwendet, da dieses Kollektiv die wissenschaftliche Standardkohorte für schwer verletzte Patienten darstellt und eine höhere Vergleichbarkeit mit der Schweizer Kohorte ermöglicht.

### Datenquellen und Zeiträume

- Deutschland: TraumaRegister DGU® (TR-DGU), Jahresbericht 2024 (Unfalljahr 2023, [1]),
- Österreich: TraumaRegister ÖGU (TR-ÖGU), Jahresbericht 2024 (Unfalljahr 2023, [2]),
- Schweiz: Da für die Schweiz derzeit keine nationalen Jahresberichte veröffentlicht werden, wurden ausschließlich publizierte STR-Analysen herangezogen, insbesondere die Arbeit von Costa et al. (2015–2019, [3, 4, 7]).

### Einschlusskriterien und Vergleichbarkeit

- Deutschland/Österreich: Das Basiskollektiv umfasst primär aufgenommene oder früh (< 48 h) zuverlegte Patienten, die entweder intensivmedizinisch versorgt werden oder mindestens eine relevante Verletzung (Abbreviated Injury Scale [AIS] ≥ 3) aufweisen. Das Basiskollektiv des TR-DGU ist definiert als alle Patienten mit mindestens einer Verletzung AIS ≥ 3 sowie Patienten mit AIS 2, die entweder intensivmedizinisch behandelt wurden oder im Krankenhaus verstorben sind. Diese Definition gilt analog für das TR-ÖGU. Eine spezifische Filterung ausschließlich auf Patienten mit Injury Severity Score (ISS) ≥ 16 ist anhand der öffentlich verfügbaren aggregierten Daten nicht möglich. Daher stellen die DE-/AT-Kollektive nicht exakt dieselbe Schweregruppe dar wie die STR-Kohorte [1, 2].
- Schweiz: Das STR (Costa et al.) umfasst ausschließlich schwer verletzte Patienten mit ISS ≥ 16 und/oder AIS des Kopfes ≥ 3. Dadurch ist die Schweizer Kohorte definitionsgemäß schwerer verletzt als die DE-/AT-Basiskollektive [4].

### Vergleichbarkeit und methodische Limitationen

Die Krankenhausmortalität im TR-DGU wird ausschließlich für jene Patienten berechnet, für die vollständige Outcome-Daten vorliegen (2023: 25.208 Patienten). Diese Outcome-Kohorte unterscheidet sich von der Zahl primär versorgter Fälle (28.718) und vom Basiskollektiv (31.217), was bei der Interpretation berücksichtigt werden muss [1].

Im TR-ÖGU hingegen wird die Mortalität direkt im Basiskollektiv berechnet (1060 Patienten) [2]. Lediglich früh weiterverlegte Patienten werden aus der Outcome-Analyse ausgeschlossen, sodass sich die zugrunde liegende Fallzahl nicht in vergleichbarem Ausmaß reduziert wie im TR-DGU.

Unterschiedliche Einschlusskriterien, Dokumentationstiefen und Zeiträume (DE/AT 2023 vs. CH 2015–2019) erlauben keine statistische Harmonisierung; die Auswertung erfolgt daher rein deskriptiv. Zusätzlich ist zu beachten, dass einzelne österreichische und schweizerische Traumazentren am TR-DGU teilnehmen und Daten doppelt gemeldet sein können; ohne Individualdaten ist eine Bereinigung potenzieller Doppelmeldungen nicht möglich.

### Analysierte Variablen

Demografische Parameter, Traumamechanismen, Verletzungsschwere (ISS, AIS), präklinische und schockraumbezogene Parameter, Intensive Care Unit (ICU-) und Krankenhausverweildauer, Mortalität sowie gesundheitsökonomische Kennzahlen, soweit verfügbar. Die gesundheitsökonomischen Daten der Schweiz basieren ausschließlich auf älteren Einzelzentrumsanalysen, da nationale Kostendaten nicht vorliegen [8, 9].

## Ergebnisse

### Deutschland – TraumaRegister DGU®

**Abb. 1:**
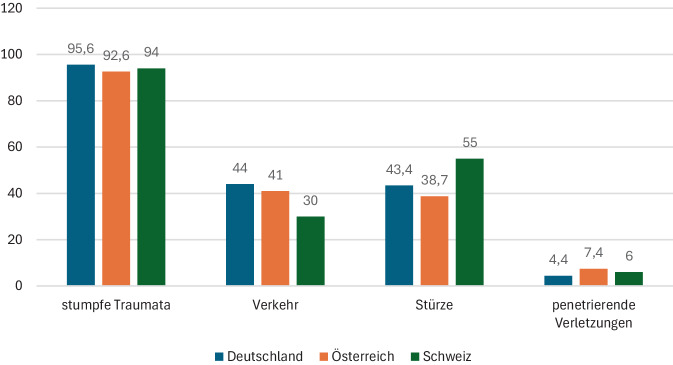
Unfallmechanismen im DACH-Raum. Dargestellt sind die Anteile der Haupttraumamechanismen aus dem TraumaRegister DGU® (Deutschland, Unfalljahr 2023, Studienkollektiv), dem TraumaRegister ÖGU (Österreich, Unfalljahr 2023) sowie dem Swiss Trauma Registry (Schweiz, 2015–2019, Costa et al. [4]). In allen 3 Ländern dominiert stumpfes Trauma (> 90 %). Während Verkehrsunfälle in Deutschland und Österreich etwa 40 % der Fälle ausmachen, sind in der Schweiz Stürze mit 55 % der häufigste Mechanismus. Penetrierende Verletzungen spielen in allen 3 Registern nur eine untergeordnete Rolle (< 10 %). Für die Schweiz werden in den STR-Publikationen keine weiteren Mechanismenkategorien ausgewiesen, was die Vergleichbarkeit der Detailmechanismen einschränkt

Das TraumaRegister DGU® wurde 1993 gegründet und zählt heute zu den größten Traumaregistern weltweit [10]. Im Jahre 2023 wurden 31.217 Patienten im Basiskollektiv erfasst, davon 28.718 primär versorgt (Ø 54,5 Jahre, 69,6 % männlich, Ø ISS 18,5). Der überwiegende Anteil der Traumata war stumpf (95,6 %), häufigste Mechanismen waren Verkehrsunfälle (39 %) und Stürze ≤ 3 m (29 %) (Tab. 1 und Abb. 1; [1]).

**Tab. 1:** Vergleich zentraler Kennzahlen polytraumatisierter Patienten im DACH-Raum. Dargestellt sind die aktuell verfügbaren Daten aus dem TraumaRegister DGU® (Deutschland, Unfalljahr 2023, Basiskollektiv), dem TraumaRegister ÖGU (Österreich, Unfalljahr 2023, Basiskollektiv) sowie den publizierten Analysen des Swiss Trauma Registry, die ausschließlich Schwerverletzte mit ISS ≥ 16 und/oder AIS des Kopfes ≥ 3 einschließen. Während die Mechanismen im STR nicht vollständig ausgewiesen werden, lassen sich stumpfe Traumata mit ~ 94 % aus der niedrigen Penetrationsrate (6 %) ableiten. Kostenangaben für Deutschland und Österreich basieren auf dem TR-DGU-Kostenschätzer (entwickelt aus realen Abrechnungsdaten 2007/2008, jährlich fortgeschrieben); schweizerische Werte beruhen hingegen auf Einzelzentrumsanalysen (u. a. Ganzoni et al. 2003 [9]). Aufgrund unterschiedlicher Einschlusskriterien, Zeiträume und Datenverfügbarkeiten ist die Tabelle rein deskriptiv zu interpretieren.

Parameter	Deutschland (TR-DGU 2023)	Österreich (TR-ÖGU 2023)	Schweiz (STR, 2015–2019)
Bevölkerung (Mio., Stand 2024)	83,5	9,2	9,03
Landesgröße (km^2^)	357.022	83.871	41.277
Registergründung (Jahr)	1993	2023	2012
Teilnehmende Kliniken (*n*)	700	33	12
Anzahl der Krankenhäuser (*n*, Stand 2023)	1874	262	275
Fälle (*n*)	28.718	2600	~ 2600/Jahr
Alter (Ø)	54,5 J.	50,2 J.	58 J.
Männer (%)	69,6 %	74 %	68 %
ISS (Ø)	18,5	22,6	22
Mechanismen – Gesamt	95,6 % stumpf/4,4 % penetrierend	92,6 % stumpf/7,4 % penetrierend	~ 94 % stumpf/6 % penetrierend
– Verkehrsunfälle	~ 44 % %	~ 41 %	30 %
– Stürze	43,4 % (≤ 3 m: 29,0 %; > 3 m: 14,4 %)	38,7 % (≤ 3 m: 18,8 %; > 3 m: 19,9 %)	55 % (meist ≤ 3 m)
ICU-Verweildauer (Ø)	6,1 Tage	9 Tage	4 Tage (Einzelzentrun Lausanne)
Liegedauer im Krankenhaus (Ø)	14,5 Tage	16,8 Tage	14,6 Tage
Mortalität im Krankenhaus (%)	7,4 % (RISC-II 8,1 %)	14,7 % (RISC-II 10,8 %)	11,6 % (4 % < 24 h)
Gesamtkosten (pro Jahr)	565 Mio. €	25 Mio. €	330 Mio. CHF
Akutkosten (Ø)	22.542 €	23.586 €	128.135 CHF (≈ 82.000 € nach Wechselkurs 2003)
Langzeitkosten (Ø)	–	–	547.800 USD (≈ 438.000 € nach Wechselkurs 2006)
Quellen	[1, 11–14]	[2, 12, 14–16]	[4, 7–9, 12, 14, 17–19]

Prähospital wurden 18 % der Patienten intubiert, 11 % befanden sich im Schock, 23 % hatten einen GCS ≤ 8 [1]. Im Schockraum erfolgte bei rund 70 % eine Ganzkörper-CT, 15–20 % erhielten eine Transfusion. Häufig betroffen waren Kopf (44 % AIS ≥ 3), Thorax (46 %), Abdomen (12 %) und Becken/Extremitäten (28 %) [1].

Die mittlere Verweildauer auf der Intensivstation (ICU) betrug 6,1 Tage, die Krankenhausliegedauer 14,5 Tage (Median 10). Die Mortalität im Krankenhaus lag bei 7,4 % in der Outcome-Kohorte (25.208 Patienten) **(**berechnet nach dem Risikoadjustierungsscore RISC-II, Revised Injury Severity Classification II: 8,1 %). Die durchschnittlichen Akutkosten betrugen laut TR-DGU 2023 22.542 € pro Patient. Für etwa 9500 dokumentierte Fälle im Kostenmodul summierten sich die Gesamtkosten auf 213,8 Mio. € [1].

### Österreich – TraumaRegister ÖGU

Das 2023 gegründete TraumaRegister in Österreich (TR-ÖGU) basiert methodisch auf dem TR-DGU und wird ab dem nächsten Jahresbericht auch einen direkten Vergleich mit Deutschland enthalten [2, 15]. Im ersten Jahresbericht wurden 1060 Patienten im Basiskollektiv erfasst (Ø 50,2 Jahre, 73,8 % männlich, Ø ISS 22,6). 92,6 % erlitten stumpfe und 7,4 % penetrierende Traumata [2].

Prähospital wurden 33 % der Patienten intubiert, 11 % befanden sich im Schock, 23 % hatten einen GCS ≤ 8. Im Schockraum erfolgte bei 83,7 % eine Ganzkörper-CT, 15 % erhielten eine Transfusion [2]. Die Verteilung der Verletzungen war mit Deutschland vergleichbar: Kopf (40 % AIS ≥ 3), Thorax (47 %), Abdomen (12 %), Becken/Extremitäten (28 %) [1, 2].

Die mittlere ICU-Verweildauer betrug 9 Tage, die Krankenhausliegedauer 16,8 Tage (Median 12). Die Mortalität im Krankenhaus lag bei 14,7 % (RISC-II: 10,8 %) (Tab. 1; [2]). Die durchschnittlichen Akutkosten beliefen sich auf 23.586 € pro Patient. Hochgerechnet auf die 1060 im TR-ÖGU erfassten Fälle würden sich damit jährliche Gesamtkosten von rund 25 Mio. € ergeben [2].

### Schweiz – Swiss Trauma Registry (STR)

Das Swiss Trauma Registry (STR) dokumentiert die Versorgung schwer verletzter Patienten in 12 zertifizierten Traumazentren in der Schweiz [7]. Eine Analyse von Costa et al. (2022) umfasste 13.222 Patienten (Ø 58 Jahre, 68 % männlich, Ø ISS 22), die zwischen 2015 und 2019 in das STR eingeschlossen wurden (ISS ≥ 16 und/oder AIS Kopf ≥ 3) [4].

Costa et al. berichten 6 % penetrierende Verletzungen; stumpfe Traumata werden nicht explizit ausgewiesen, können jedoch mit rund 94 % aus der geringen Penetrationsrate abgeleitet werden. Als Verletzungsmechanismen werden 55 % Stürze, 30 % Verkehrsunfälle und 2 % Schuss‑/Stichverletzungen angegeben; weitere Mechanismen werden nicht separat ausgewiesen [4]. Der mittlere ISS betrug 22 Punkte, bei massiv transfundierten Patient:innen 32. Die Gesamtletalität betrug 11,6 %, wobei 4 % der Patienten innerhalb der ersten 24 h verstarben [4].

Daten zur intensivmedizinischen Versorgung liegen nur aus Einzelanalysen vor. Eine Auswertung des Lausanner Traumaregisters (2005–2010) berichtete eine mittlere ICU-Verweildauer von 4 Tagen und eine mittlere Krankenhausverweildauer von 7 Tagen [17].

Gesundheitsökonomische Daten liegen nur in Einzelfallanalysen vor. Eine Untersuchung am Universitätsspital Zürich (Ganzoni et al. [9]) bezifferte die durchschnittlichen Akutkosten bei schwer polytraumatisierten Patienten (Ø ISS 33,9) mit 128.135 CHF pro Fall ≈ 82.000 € nach durchschnittlichem Wechselkurs 2003 (1 CHF ≈ 0,64 €) [9]. Haeusler et al. (2006) berechneten in einer Langzeitanalyse durchschnittliche Lebenszeitkosten von 547.800 USD (≈ 438.000 € nach Wechselkurs 2006 = 1 USD ≈ 0,80 €), wobei zwei Drittel auf Produktivitätsverluste entfielen [8].

## Diskussion

### Vergleichende Betrachtung im DACH-Raum

Diese Arbeit vergleicht erstmals systematisch die Traumaregister im DACH-Raum anhand der 3 nationalen Register Deutschland (TR-DGU), Österreich (TR-ÖGU) und Schweiz (STR). Die Analyse zeigt insgesamt eine hohe Versorgungsqualität in allen 3 Ländern, weist jedoch klare Unterschiede in Altersstruktur, Mechanismen und Mortalität auf. Tab. 1 fasst die wichtigsten Kennzahlen zusammen.

Die methodische Vergleichbarkeit ist insbesondere zwischen TR-DGU und TR-ÖGU hoch, während das STR aufgrund der strengeren Einschlusskriterien (ISS ≥ 16/AIS des Kopfes ≥ 3) ein schwerer verletztes Patientenkollektiv abbildet [1, 2, 4]. Für Deutschland ist über mehrere Jahrgänge hinweg eine hohe Stabilität zentraler Parameter nachweisbar, was auf robuste Dokumentations- und Qualitätsprozesse im TR-DGU hinweist [1, 20].

### Gemeinsamkeiten und Unterschiede in Epidemiologie und Mechanismen

Obwohl die Register unterschiedliche Entstehungshintergründe und Datenverfügbarkeiten aufweisen, ist ein direkter Vergleich zentraler Parameter möglich. Deutschland und Österreich nutzen nahezu identische Erfassungs- und Definitionsstandards, während das Schweizer Trauma-Register (STR) auf derselben methodischen Grundlage basiert, jedoch als behördlich angeordnete Qualitätskontrolle organisiert ist [7, 11, 15]. Dadurch stehen keine vollständigen nationalen Jahresberichte zur Verfügung, was die Vergleichbarkeit einschränkt. Die hier ausgewerteten Schweizer Daten (2015–2019) basieren auf den bislang publizierten STR-Analysen [4, 17].

Die Auswertung zeigt deutliche Gemeinsamkeiten, aber auch länderspezifische Unterschiede. In allen 3 Ländern dominiert das stumpfe Trauma mit einem Anteil von über 90 % (Tab. 1). Verkehrsunfälle und Stürze sind die Hauptursachen, wobei in der Schweiz ein deutlich höherer Anteil an Low-Energy-Trauma durch Stürze aus geringer Höhe auffällt, was auf die dort ältere Patientenpopulation zurückzuführen ist.

### Demografische Unterschiede und geriatrisches Polytrauma

Ein wesentlicher Unterschied betrifft die Altersstruktur. Schweizer Patienten sind im Mittel älter (58 Jahre) als Patienten in Deutschland (54,5 Jahre) und Österreich (50,2 Jahre) [1, 2, 4]. Dies könnte den höheren Anteil an Stürzen erklären, insbesondere Low-Energy-Traumata, in der Schweiz (55 %) im Vergleich zu Deutschland (29 % Stürze ≤ 3 m) und Österreich (ca. 30 %) [1, 2]. Diese Entwicklung reflektiert den demografischen Wandel und verdeutlicht die zunehmende Bedeutung geriatrischer Traumata im DACH-Raum. In allen 3 Ländern muss die Versorgung künftig noch stärker auf diese Patientengruppe ausgerichtet werden, da Komorbiditäten, eingeschränkte physiologische Reserven und häufige Antikoagulation besondere Anforderungen an Ressourcen, perioperatives Management und Outcome-Prognosen stellen.

### Mortalitätstrends und Risikoadjustierung

Die Krankenhausletalität variiert im Rohvergleich zwischen den Ländern. Im Studienkollektiv des TR-DGU lag die Krankenhausletalität bei 7,4 %, während sie im TR-ÖGU 14,7 % und im STR 11,6 % betrug [1, 2, 4]. Damit wurden vergleichbare Patientengruppen gegenübergestellt, da in allen Registern primär versorgte Schwerverletzte (ISS ≥ 16 bzw. AIS des Kopfes ≥ 3) berücksichtigt wurden. Die höhere Letalität im Basiskollektiv des TR-DGU (13 %), das auch Sekundärverlegungen und unvollständig dokumentierte Fälle enthält, wurde in diesem Vergleich nicht berücksichtigt. Nach Risikoadjustierung (RISC-II) gleichen sich die Ergebnisse weitgehend an, sodass die Unterschiede eher durch Patientenselektion, Unterschiede in der Patientencharakteristik und kleinere Kollektive erklärbar sind als durch echte Versorgungsdefizite. Ein direkter statistischer Vergleich ist mit den publizierten Registerdaten nicht möglich, da Konfidenzintervalle und Individualdaten fehlen. Formale Signifikanztests oder Box-Plots wären nur mit harmonisierten DACH-Individualdaten möglich.

Langzeitdaten zeigen, dass sich die Krankenhausletalität in Deutschland in den letzten 2 Jahrzehnten von über 15 % auf derzeit rund 7 % halbiert hat [1, 10]. Dieser Rückgang ist Ausdruck wesentlicher Fortschritte in der Polytraumaversorgung, insbesondere im Schockraummanagement, in der standardisierten Bildgebung (Ganzkörper-CT) und in der Implementierung der S3-Leitlinie Polytrauma. Damit bestätigt sich, dass strukturierte Behandlungsstandards und interdisziplinäre Prozesse entscheidend zur Verbesserung des Outcomes beigetragen haben.

### Präklinische Versorgungssysteme

Die präklinische Versorgung unterscheidet sich strukturell deutlich zwischen den Ländern. Deutschland und Österreich setzen überwiegend auf ein notarztbasiertes System, während in der Schweiz ein Paramedic-orientiertes Modell etabliert ist [3]. Dies zeigt sich u. a. in den Intubationsraten (DE 18 %, AT 31 %; publizierte Schweizer STR/TR-DGU-Analysen ≈ 31 %). Trotz dieser strukturellen Unterschiede sind zentrale Prozessparameter wie Schockraum-CT-Raten (DE ~70 %, AT ~84 %) und Transfusionsraten (DE 15–20 %, AT 15 %) vergleichbar.

Jensen et al. zeigten, dass Unterschiede im präklinischen Vorgehen zwischen notarzt- und Paramedic-basierten Systemen bestehen, diese sich jedoch nach Risikoadjustierung nicht in einem Mortalitätsunterschied widerspiegeln [3]. Ergänzend berichten Magyar et al. für schwer penetrierte Verletzungen längere prähospitale Einsatzzeiten in der Schweiz (Median 65 min), was strukturelle und geografische Faktoren reflektiert [21]. Insgesamt deuten diese Daten darauf hin, dass die Systemstruktur das präklinische Vorgehen beeinflusst, die Auswirkungen auf das Überleben jedoch gering sind.

### Intensiv- und Krankenhausversorgung

Die Krankenhausaufenthaltsdauer ist in Österreich mit 16,8 Tagen am längsten, gefolgt von der Schweiz (14,6 Tage) und Deutschland (14,5 Tage). Auch die intensivmedizinische Behandlung variiert. Österreich berichtet mit durchschnittlich 9 Tagen die längste ICU-Verweildauer, während Deutschland bei 6,1 Tagen liegt [1, 2]. Für die Schweiz werden in einer Einzelzentrumsanalyse aus Lausanne mittlere ICU-Verweildauern von 4 Tagen beschrieben [17], repräsentative nationale STR-Daten liegen hierzu bislang jedoch nicht vor. Unterschiede könnten auf Ressourcenverfügbarkeit, Patientenschwere oder Entlassungsstrategien zurückzuführen sein, bedürfen jedoch weiterer Analysen.

### Ökonomische Bewertung

Gesundheitsökonomisch liegen die durchschnittlichen Akutkosten in Deutschland (22.542 €) und Österreich (23.586 €) auf vergleichbarem Niveau [1, 2]. Für die Schweiz zeigte eine Untersuchung am Universitätsspital Zürich deutlich höhere Akutkosten von 128.135 CHF pro Patient [9]. Darüber hinaus wurde 2006 in einer Langzeitanalyse (Haeusler et al.) ein durchschnittlicher lebenslanger Aufwand von 547.800 USD pro Patient berechnet, wobei zwei Drittel der Kosten auf indirekte Kosten wie Arbeitsausfall und Invalidität entfielen [8]. Damit ist die Schweiz das einzige DACH-Land mit publizierten Langzeitkostendaten. Während die Angaben für Deutschland und Österreich auf modellhaften Kostenschätzungen basieren, liegen für die Schweiz reale Abrechnungsdaten aus Einzelanalysen vor [9]. Diese Unterschiede erschweren direkte Vergleiche und verdeutlichen den Bedarf an harmonisierten ökonomischen Erfassungsstandards.

Die Kostenschätzung beruht dabei nicht auf realen Einzelabrechnungen aller Patienten, sondern auf einem validierten Kostenschätzer, der ursprünglich anhand von 1002 Patienten mit vollständigen Abrechnungsdaten aus den Jahren 2007/2008 entwickelt wurde [22]. In dieser Analyse wurde ein Rechenmodell erstellt, das mithilfe weniger Variablen (ISS, ICU- und Beatmungsdauer, Normalstationstage, Blutprodukte, Verletzungsmuster) die tatsächlichen Behandlungskosten mit hoher Genauigkeit (Abweichung < 3 %) abbilden konnte. Seitdem wird dieses Modell im TR-DGU für alle dokumentierten Fälle angewendet, wobei die Referenzwerte unter Annahme einer jährlichen Kostensteigerung von 2 % fortgeschrieben werden [22].

Hochgerechnet auf die 25.208 im TR-DGU erfassten Fälle ergeben sich damit jährliche Gesamtkosten von rund 565 Mio. € [1]. Hier sind allerdings auch nur die direkten Kosten der Akutversorgung erfasst ohne Berücksichtigung der indirekten Kosten (Arbeitsunfähigkeit, Rehabilitationskosten, Frührente, etc.). In Deutschland und Österreich fehlen bislang vergleichbare Analysen, sodass der wahre gesellschaftliche Aufwand vermutlich unterschätzt wird. Summiert man die jährlichen direkten Akutkosten aller 3 Länder, ergeben sich für den DACH-Raum Gesamtaufwendungen von rund 905 Mio. € pro Jahr, wobei die indirekten Kosten diese Summe um ein Vielfaches übersteigen dürften.

## Limitationen

Die Vergleichbarkeit der Register ist durch unterschiedliche Einschlusskriterien und Datenverfügbarkeiten eingeschränkt. Während TR-DGU und TR-ÖGU vollständige nationale Jahresberichte liefern, stehen für die Schweiz keine öffentlichen STR-Jahresberichte zur Verfügung. Die Auswertung basiert daher ausschließlich auf publizierten STR-Studien, die nur Teilkollektive abbilden und sich hinsichtlich Zeitraum und Methodik unterscheiden [5, 12]. Insbesondere intensivmedizinische Parameter liegen für die Schweiz nur aus Einzelzentrumsanalysen vor, was ihre Repräsentativität limitiert. Ein direkter statistischer Vergleich zwischen den Ländern ist aufgrund fehlender Individualdaten und nichtverfügbarer Konfidenzintervalle nicht möglich. Potenzielle Doppelmeldungen können nicht ausgeschlossen werden, da einige österreichische und schweizerische Zentren parallel am TR-DGU teilnehmen. Diese Faktoren müssen bei der Interpretation berücksichtigt werden.

## Ausblick

Trotz dieser Einschränkungen unterstreicht die Übersicht die zentrale Bedeutung von Registerdaten für Forschung und Qualitätssicherung. Während damals nur fragmentarische Daten vorlagen und v. a. prähospitale Unterschiede zwischen notarzt- und Paramedic-basierten Systemen diskutiert wurden [3], stehen heute mit TR-DGU, TR-ÖGU und STR im DACH-Raum 3 leistungsfähige und methodisch robuste Datenquellen zur Verfügung. Eine integrative DACH-weite Analyse fehlt jedoch bislang. Eine engere Kooperation zwischen den Registern könnte mittelfristig zu gemeinsamen Auswertungen führen, Synergieeffekte schaffen und die internationale Sichtbarkeit erhöhen.

## Fazit

Nach Risikoadjustierung zeigen die Register eine vergleichbare Versorgungsqualität in Deutschland, Österreich und der Schweiz. Unterschiede in Patientenprofil, Traumamechanismus und Ressourcennutzung unterstreichen den Bedarf länderspezifischer, altersadaptiver Versorgungskonzepte. Eine engere Vernetzung der Register mit harmonisierten Datensätzen könnten künftig zu einer verbesserten Evidenzbasis und gemeinsamen Versorgungsstrategien beitragen.

## Fazit für die Praxis

- Ein Polytrauma bleibt eine der größten Herausforderungen für Unfallchirurgie, Notfall- und Intensivmedizin im DACH-Raum.
- Registerdaten aus Deutschland (TR-DGU), Österreich (TR-ÖGU) und die publizierten STR-Analysen ermöglichen eine valide Analyse von Epidemiologie, Outcome und Versorgungsqualität.
- Gemeinsamkeiten: hoher Anteil stumpfer Traumata (> 90 %), überwiegend männliche Patienten, Verkehrsunfälle und Stürze als Hauptmechanismen.
- Unterschiede: Schweizer Patienten sind älter, Stürze aus geringer Höhe häufiger; Österreich zeigt höhere Mortalität und längere ICU-Verweildauern.
- Gesundheitsökonomische Daten liegen v. a. aus Deutschland und Österreich vor (~22.000–24.000 € pro Patient); für die Schweiz besteht Forschungsbedarf.

## Data Availability

Die in dieser Studie analysierten Daten stammen aus öffentlich zugänglichen Quellen, einschließlich des Jahresberichts des TraumaRegister DGU®, des Jahresberichts des TraumaRegister ÖGU sowie aus publizierten Analysen des Swiss Trauma Registry. Es wurden ausschließlich anonymisierte und aggregierte Daten verwendet. Es wurden keine Individualdaten erhoben oder analysiert. Die entsprechenden Datenquellen sind im Manuskript zitiert und in den Referenzen aufgeführt.
